# Blood Coagulation Favors Anti-Inflammatory Immune Responses in Whole Blood

**DOI:** 10.3390/hematolrep17020019

**Published:** 2025-04-11

**Authors:** Victor I. Seledtsov, Anatoly A. Pyshenko, Tatyana Ya. Lyubavskaya, Irina A. Seledtsova, Alexei A. von Delwig

**Affiliations:** Federal State Budgetary Scientific Institution, Russian Scientific Center for Surgery Named after Academician B.V. Petrovsky, 119991 Moscow, Russia; anatoliy.dr@yandex.ru (A.A.P.); rnc2016@mail.ru (T.Y.L.); iax34@yandex.ru (I.A.S.); delvig59@mail.ru (A.A.v.D.)

**Keywords:** blood coagulation, whole blood immunoreactivity, reactive oxygen species, cytokines, regeneration

## Abstract

Background: We studied the effects of human blood coagulation on antioxidant activity and the cellular secretion of immunoregulatory molecules in vitro. Methods: Reactive oxygen species (ROS) activity and cytokine content were determined in plasma and serum blood samples incubated with lipopolysaccharide (LPS) for 3 h or 18 h. Results: Coagulation process significantly decreased ROS activity induced by LPS in blood samples from healthy donors. Human serum was found to have significantly higher antioxidant activity than plasma. Blood coagulation markedly reduced LPS-induced secretion of TNF-α by cells, without significantly affecting the secretion of interleukin-1 (IL-1), IL-6, IL-8, or C-reactive protein (CRP). Blood clotting led to an increase in LPS-induced release of vascular endothelial growth factor (VEGF) by blood cells. A significant increase in procalcitonin levels was also observed in serum samples. Conclusions: Blood clotting enhances the antioxidant and anti-inflammatory functions of immunoreactive blood cells.

## 1. Introduction

Homeostasis and the immune system are closely interconnected, relying on shared pathways and regulatory processes. This relationship is crucial for defending against injuries and infections; however, when dysregulated, it can contribute to disease. Therefore, studying the control mechanisms of both blood coagulation and immune reactivity is of pivotal importance.

The primary role of blood coagulation is to maintain consistent blood flow to the tissues. Additionally, it substantially enhances interactions between platelets and leukocytes, which are crucial for blocking pathogen invasion [1]. Platelet activation is accompanied by the release of numerous immunoactive and growth factors, such as interleukin-1 (IL-1), platelet-derived growth factor (PDGF), insulin-like growth factor-1 (IGF-1), vascular endothelial growth factor (VEGF), platelet-derived angiogenic factor (PDAF), transforming growth factor-beta (TGF-β), chemokines, and prostaglandins. These factors contribute to linking blood coagulation with regeneration and immune functions [1,2,3]. Additionally, activated platelets express immunoactive co-stimulatory molecules, such as CD40L (CD154), further linking blood clotting with the activation of innate and adaptive immune cells [1]. To control excessive inflammatory responses, large amounts of biologically active interleukin-1 receptor antagonist (IL-1RA) and other cytokines (IL-4, IL-6, IL-10, IL-13, IL-17, IL-22) have been shown to be released during the blood coagulation process [4,5]. This suggests that coagulation could regulate both local and systemic immune responses to inflammatory stimuli and create the necessary conditions for regenerative processes, even in the presence of infection [6].

In this study, we examined the early, direct effects of the human blood coagulation process on the immunobiological reactivity of blood cells within the first few hours after clot formation begins.

## 2. Materials and Methods

The study was approved by the local ethics committee of the Petrovsky National Research Centre of Surgery (protocol N° 9, October 2024). All blood donors were informed about the use of their blood for research purposes, and informed consents were obtained.

### 2.1. Collection of Whole Blood Samples

Whole blood samples were collected from 23 healthy donors (aged 25–45) into standard cold vacutainer tubes, either with or without heparin. The blood was then divided into 1 mL and 2 mL samples and stored on ice for no more than 2 h before use.

### 2.2. Measurements of Reactive Oxygen Species (ROS)

Venous blood samples (1 mL each), with or without heparin, were incubated at 37.5 °C for 30 min. Lipopolysaccharide (LPS) from Salmonella typhi (50 μL, Pyrogenal, Med-gamal, Moscow, Russia) was then added to the blood at a final concentration of 1 µg/mL, or saline was added as a control. All samples were treated with a 50 μL aliquot of 1M luminol solution (Sigma-Aldrich, St. Louis, MO, USA) at the same time. Both LPS and control samples underwent a two-hour incubation at 37.5 °C. ROS activity in the blood samples reached a steady state during this period before decreasing. ROS concentrations were determined in the samples based on the chemiluminescence intensity measured using a Lum-1200 luminometer (LLC DISoft, Moscow, Russia), following the manufacturer’s instructions. The PowerGraph software package (version 3.3, DISoft Company, Moscow, Russia) was used for data acquisition, signal logging, real-time visualization and processing, as well as for data editing, mathematical processing, and analysis.

Blood samples were incubated at 37.5 °C for 3 h, with or without heparin, to obtain plasma and serum, respectively. Centrifugation at 2000× *g* for 20 min was used to separate the liquids from the cells. Saline solution was ozonized by passing it through a medical ozone generator (Medozons-BM, Nizhny Novgorod, Russia) and used immediately. Blood plasma or serum samples (1 mL) were incubated with ozonized saline (1 mL) or 3% hydrogen peroxide (5 μL), along with 1M luminol solution, at 37.5 °C for 2 h. ROS levels in the samples were measured as described above.

### 2.3. Assaying Bioactive Molecules

Blood samples (2 mL each) were incubated for 3 or 18 h at 37 °C with or without heparin, in the presence or absence of LPS. Concentrations of IL-1, IL-6, IL-8, TNF-α, CRP, procalcitonin, and VEGF were measured in serum and plasma samples using commercially available ELISA kits from Vector-Best (Novosibirsk, Russia), following the manufacturer’s instructions.

### 2.4. Statistical Analysis

Significant differences within paired blood samples were analyzed using the paired Student’s *t*-test. Data in the figures are presented as means ± their errors. Differences were considered significant at *p* < 0.05.

## 3. Results

### Antioxidant Effect of Blood Clotting

**Figure 1:**
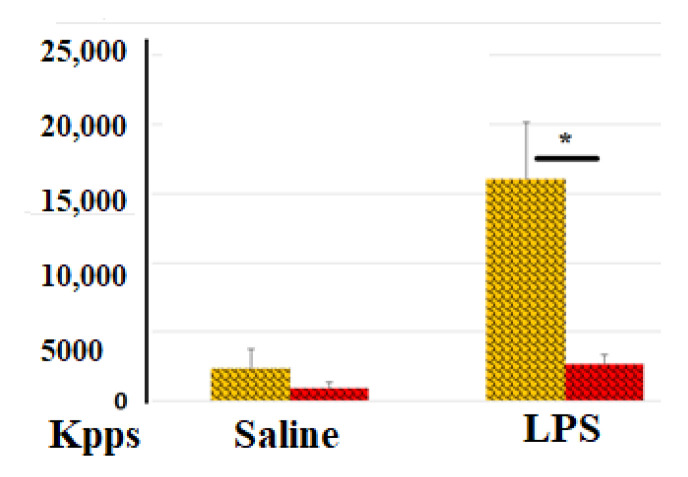
Effect of blood coagulation on ROS activity in blood samples. ROS activity (total number of kilopockets in photon mode, Kpps) in blood samples incubated with (yellow columns) or without heparin (red columns) in the absence or presence of LPS. The results from identical experiments with blood samples from 4 donors (n = 4) are shown. * Differences are statistically significant (*p* < 0.05).

Changes in ROS production are among the most immediate responses of blood cells to environmental stimuli. ROS (O2− (superoxide), H2O2 (hydrogen peroxide), OH- (hydroxyl radical), 1O2 (singlet oxygen), and α-O (alpha-oxygen)) trigger chain reactions that can lead to DNA/RNA damage, protein oxidation, and lipid (per)oxidation [7]. Infection is well known to be a potent stimulus for ROS production by immune cells. Thrombosis, along with associated inflammation (thromboinflammation), also involves leukocytes, platelets, and endothelial cells, leading to the production of ROS in the bloodstream. In our experiments, we used LPS-treated blood cells to model the effects of an infectious agent that typically accompanies blood vessel trauma. It was of interest how the process of blood coagulation, even at the earliest stages of its development, can influence ROS secretion by blood cells. Figure 1 demonstrates that LPS-induced ROS activity was significantly reduced (6-fold lower) in samples with clotted blood compared to those with non-clotted blood.

**Figure 2:**
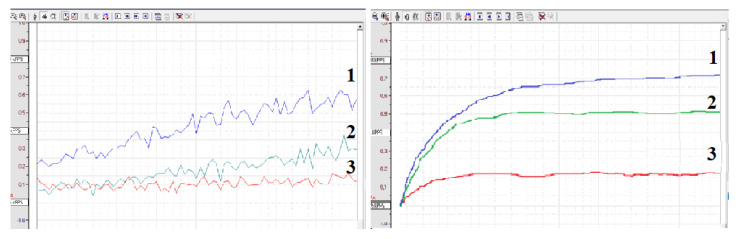
Chemiluminescence intensity of ozonized saline diluted 2-fold with physiological solution (1), plasma (2) or serum (3).

**Figure 3:**
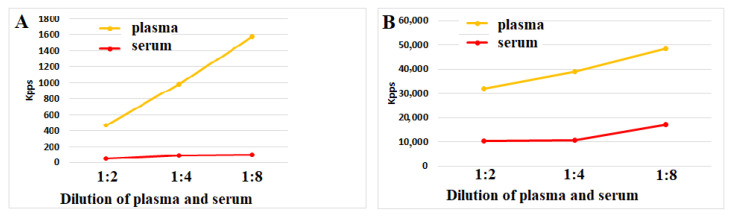
(**A**,**B**) ROS activity (Kpps) induced by ozonized saline solution (**A**) or hydrogen peroxide (**B**) in plasma or serum samples. Control values for ozonized saline and hydrogen peroxide solutions in the absence of plasma/serum were 2850 and 61,870 Kpps, respectively. The representative results from identical experiments with samples from 4 donors are shown.

This phenomenon can be explained, at least in part, by the increased antioxidant activity of serum. Figure 2 and Figure 3A,B show that both plasma and serum can reduce ROS activity upon incubation with ozonized saline or hydrogen peroxide. Notably, serum antioxidant activity consistently and significantly exceeded plasma levels in all four experiments (n = 4) at every dilution. Our findings suggest that blood coagulation is associated with reduced ROS-mediated reactivity of immune blood cells. This effect may be partially explained by increased antioxidant activity in serum compared to plasma.

#### Anti-Inflammatory Effects of Blood Clotting

**Figure 4:**
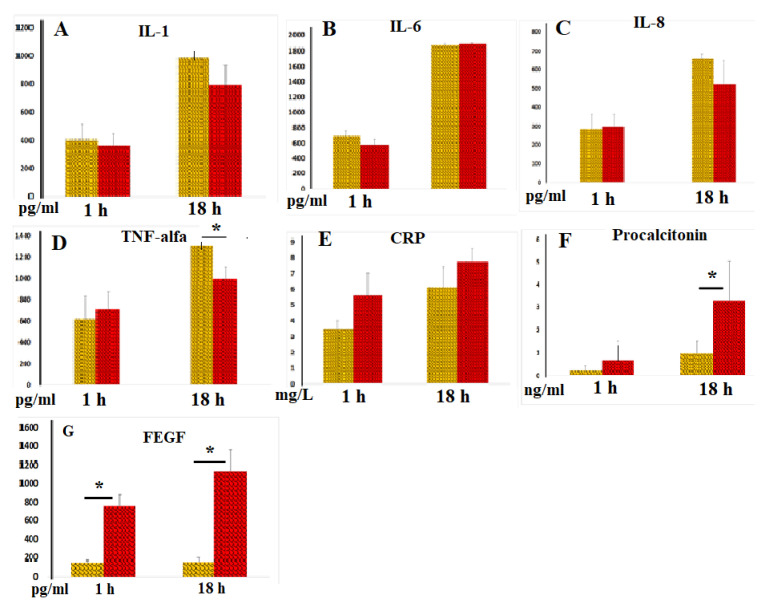
(**A**–**G**) Concentrations of IL-1 (**A**, n = 5), IL-6 (**B**, n = 5), IL-8 (**C**, n = 5), TNF-α (**D**, n = 7), CRP (**E**, n = 7), procalcitonin (**F**, n = 7), and FEGF (**G**, n = 5) in plasma (yellow columns) and serum samples (red columns) following 3 and 18 h of incubation with LPS. * *p* < 0.05.

IL-1, IL-6, IL-8, and TNF-α are key mediators that trigger inflammation [8]. As expected, incubation of blood with LPS resulted in a dramatic enhancement of cellular IL-1 secretion. Figure 4A demonstrates that IL-1 levels in sera (in the samples without heparin) after both 3 and 18 h of blood incubation were similar to those observed in plasma (in samples with heparin). In control samples without LPS, low IL-1 concentrations (<30 pg/mL) were detected, which were nearly identical in serum and plasma samples.

In the absence of LPS blood stimulation, both plasma and serum samples exhibited low IL-6 concentrations (<50 pg/mL). When blood was incubated with LPS, IL-6 secretion increased dramatically. No significant differences were observed between IL-6 levels in plasma and serum (Figure 4B).

LPS moderately increased IL-8 release from blood cells; plasma and serum IL-8 levels were similar (Figure 4C).

The TNF-α content in blood samples without LPS did not exceed 100 pg/mL. As expected, LPS dramatically increased TNF-α secretion by blood cells. After 18 h of incubation, TNF-α concentrations in serum samples were significantly lower than those in plasma samples. However, after 3 h of incubation of blood with LPS, similar levels of this key pro-inflammatory cytokine were observed in both serum and plasma samples (Figure 4D), suggesting that blood coagulation acquired substantial anti-inflammatory potential only 18 h after coagulation began.

C-reactive protein (CRP) and procalcitonin are well-established markers of systemic inflammation. The CRP content in blood samples without LPS did not exceed 1.5 mg/L. In our experiments, we observed significant increases in CRP secretion only after 18 h of incubation of blood with LPS, with no stable significant differences noted between serum and plasma samples (Figure 4E). However, in some experiments, there was a tendency for higher CRP concentrations in serum compared to plasma samples. We also measured procalcitonin levels and found that, after 18 h of blood incubation with LPS, the concentrations of this peptide precursor of calcitonin and marker of bacterial infection were threefold higher than those in plasma samples (Figure 4F). These data suggest that blood coagulation enhances procalcitonin secretion by blood cells.

The primary function of VEGF is to promote the formation of new blood vessels after trauma and facilitate collateral blood supply (i.e., the development of new vessels when existing ones are blocked). The results of our experiments indicated that VEGF concentrations in intact serum samples were noticeably elevated compared to intact plasma samples Statistically significantly higher levels of VEGF were also detected in serum samples compared to plasma samples after 3 and 18 h of blood incubation with LPS (Figure 4G). Notably, LPS exhibited only a relatively weak stimulatory effect on cellular VEGF secretion, highlighting the powerful regenerative potential of the blood coagulation process.

## 4. Discussion

Blood clotting is essential for maintaining the organism’s integrity by forming a barrier that prevents blood from leaking out of the bloodstream. Normally, when blood vessels are damaged, endothelial cells and blood cells—primarily monocytes and macrophages—upregulate the production of the transmembrane protein tissue factor (TF). The interaction between TF and circulating blood-coagulation factors triggers the coagulation cascade, leading to fibrin clot formation, effectively linking blood clotting to inflammation [2,9]. Furthermore, blood clotting serves a twofold purpose, occurring simultaneously or sequentially: (i) preventing pathogens from spreading within the body and (ii) providing the necessary conditions for tissue repair.

ROS are critical for protecting against microbial and cancer dangers [7]. On the other hand, they are primarily responsible for inflammation-driven damage to cells and tissues, thus accelerating body aging and causing diseases [10]. Therefore, ROS generation and activity are strictly controlled in the body. ROS are pivotal in promoting the expression of genes encoding antioxidant enzymes (such as superoxide dismutase, glutathione peroxidase, glutathione S-transferase, catalase, haem oxygenase-1, NADPH–quinone oxidoreductase, heat shock proteins, etc.), which protect against oxygen-related cellular damage [10]. In this report, we present evidence that blood coagulation may lead to decreased ROS activity in blood samples and that this effect may be mediated, at least in part, by increased cellular secretion of antioxidant molecules. These biomolecules are primarily needed to mitigate ROS-induced damage to healthy tissues next to the blood clot.

Platelets are known to interact with immune cells, with about 3% of lymphocytes physically linked to platelets. Platelet activation increases their association with lymphocytes [1,11], while lymphocyte activation also promotes direct platelet-lymphocyte interactions [12,13]. This platelet-lymphocyte cross-talk stimulates the generation of pro-inflammatory Th1 and Th17 cells [14,15], establishing an important regulatory mechanism in thrombosis, inflammation, immunity, and atherosclerosis [12,13,16]. Our data demonstrated that blood clotting significantly enhanced IL-8 and VEGF secretion by blood cells in the absence of LPS. This suggests that the coagulation process, even in its early stages, attracts immune cells—primarily neutrophils—to sites of tissue damage and helps prevent ischemic tissue damage.

Injury to blood vessels raises the risk of microbial pathogens entering the body. LPS, a mandatory component in all Gram-negative bacteria, strongly activates innate immunity. The binding of LPS by TLR4 and CD14 on immune cells also determines the potency and Th1/Th2 polarization of adaptive immune reactions [17]. Blood clots have been shown previously to effectively bind LPS, thus influencing LPS-dependent immune processes within the clot [18]. We designed experiments where whole blood was incubated with LPS to model the simultaneous effects of an infectious agent and blood coagulation on immunogenesis. We stress that this experimental model closely resembles natural conditions, with no synthetic culture medium used, except for heparin, which was added to inhibit blood coagulation. With this model, we examined how coagulation impacts the release of pro-inflammatory cytokines IL-1, IL-6, IL-8, and TNF-α, which can be involved in both protective immune responses and severe life-threatening conditions caused by immune dysregulation. The selection of these cytokines was also based on their known pro-coagulant effects. Indeed, IL-1 upregulates TF expression in endothelial cells, monocytes, and other blood cells; IL-6 stimulates fibrinogen production; IL-8 recruits neutrophils to sites of tissue damage, activating their pro-coagulant properties; and TNF-α activates platelets and promotes their aggregation [8,19]. Taken together, the cytokines selected and analyzed in our study can be considered key players in the omnidirectional interactions between the homeostasis system and the immune system, both in normal and pathological conditions.

Our data showed that LPS stimulated the production of all pro-inflammatory cytokines studied, both in the presence and absence of heparin. Cytokine production was detected as early as within the initial 3 h of LPS exposure, reaching its peak at 18 h. Under these conditions, blood coagulation substantially reduced TNF-α production from cells stimulated by LPS, while levels of IL-1, IL-6, IL-8, and CRP were unaffected. Previously, it was shown that the formation of a blood clot stimulates the secretion of various factors by blood cells, including IL-10 and IL-13, which have strong anti-inflammatory properties [4]. In light of the available data, we envisage that even in the presence of an infectious agent, blood coagulation-mediated immunoregulation is primarily aimed at containing inflammatory cytodestructive processes and creating the necessary conditions for the development of regenerative processes. To support this assumption, our experiments also revealed that blood coagulation enhanced both spontaneous and LPS-induced VEGF secretion by blood cells, suggesting that increased VEGF secretion, along with reduced ROS levels in situ, could promote neoangiogenesis at tissue damage sites, favoring tissue regeneration.

Interestingly, sera in blood samples exposed to LPS showed higher procalcitonin levels than plasmas. Elevated procalcitonin in the blood is known to be an indicator of a bacterial infection in the body. These observations support the idea that bacterial infections, which cause more severe blood vessel damage compared to viral infections, may more effectively activate blood coagulation, subsequently stimulating procalcitonin secretion by blood cells.

## 5. Conclusions

The data from this study suggest that the blood coagulation process not only forms a barrier preventing blood from leaving the vascular channel, but also promotes antioxidant, anti-inflammatory, and regenerative cellular reactivities essential for repairing damaged tissue structures. Bacterial products, such as LPS, may serve as additional mobilizing factors in this regard.

## Data Availability

Permission is not required to share data.
